# Laser projector method for measuring postoperative acetabular anteversion after total hip replacement

**DOI:** 10.3389/fsurg.2022.1033453

**Published:** 2022-10-24

**Authors:** Wei-Cheng Chen, Tai-Yin Wu, Kuan-Yu Chi, Pei-Wei Weng, Yu-Min Huang, Yi-Chia Lin, Chen-Kun Liaw

**Affiliations:** ^1^ Department of Orthopedics, Shuang Ho Hospital, Taipei Medical University, New Taipei City, Taiwan; ^2^ Department of Family Medicine, Taipei City Hospital, Taipei, Taiwan; ^3^ Institute of Epidemiology and Preventive Medicine, National Taiwan University, Taipei, Taiwan; ^4^ Department of Internal Medicine, Taipei Medical University Hospital, Taipei, Taiwan; ^5^ Department of Education, Center for Evidence-Based Medicine, Taipei Medical University Hospital, Taipei, Taiwan; ^6^ Glickman Urologic and Kidney Institute, Cleveland Clinic, Cleveland, OH, United States; ^7^ Department of Orthopedics, School of Medicine, College of Medicine, Taipei Medical University, Taipei, Taiwan; ^8^ Department of Orthopedics, National Taiwan University Hospital, Taipei, Taiwan

**Keywords:** total hip replacement, total hip arthroplasty, computer aided (CA) systems, acetabular anteversion (AA), laser projecting system

## Abstract

**Introduction:**

For patients undergoing THR, measuring the postoperative acetabular anteversion precisely plays a pivotal role in the prognosis. However, using elliptical methods mandates computerized equipment that is frequently in shortage in remote areas and developing countries. We invented a laser projector utilizing the ellipse method to measure the acetabular anteversion directly. The aim is to examine the consistency and validity of the laser projector as compared to our original software, Elliversion.

**Materials and Methods:**

We retrospectively collected 50 postoperative pelvis radiographs including acetabulum from our institution. One investigator first measured the anteversion of included radiographs through Elliversion software as the control group. Subsequently, two operators independently used the laser projector for measurements in two separate periods with 1-day intervals as the experimental group. Our analysis was comprised of intra- and inter-observer comparisons and reliability, which investigated both the consistency and validity, by using two-sample student's *t*-test and intraclass correlation coefficient.

**Results:**

There was no significant difference in measuring the anteversion through laser projectors between two operators (*p* = 0.54), with excellent inter-observer reliability (ICC, 0.967). The estimated effect in the anteversion measurement between the Elliversion and laser projector was also comparable, with the ICC level of 0.984, indicating excellent reliability.

**Conclusion:**

Our study reported the consistency and validity of this laser projector as there is no significant difference between Elliversion and Laser projector, notably with excellent intra- and inter-observer reliability. We look forward to helping elevate clinical acumen when doctors provide care to patients after THR, especially in remote areas.

## Strengths and limitations of this study

We invented a portable laser projector method utilizing the elliptical method for orthopedic surgeons to feasibly and conveniently measure postoperative acetabular anteversion to help elevate clinical acumen when doctors provide care to patients after total hip replacement, especially in remote areas. However, as the Elliversion, the precision of anteversion measurement would be hindered by the quality of plain radiography despite laser projector method being operator-independent.

## Introduction

the most common elective operations in orthopedic field and it has been predicted that its annual volume will spike up to 572,000 by 2030 (1). For patients undergoing THR, measuring the postoperative acetabular anteversion precisely plays a pivotal role in the prognosis because anteversion of acetabulum cup determines the range of motion and stability after the THR (2). To date, the documented techniques for assessing anteversion could be classified into two-dimensional (2D) and three-dimensional (3D) methods. 2D methods included the trigonometric (3–5), the protractor (2, 6–10), and the computerized ellipse (11) method. The computerized tomography (12) (CT) method is representative of 3D method, which pertains an excellent solution but is clinically unpractical due to its high cost and risk of radiation exposure. Regarding the 2D methods, we have already validated the protractor and computerized ellipse methods and concluded that exploiting computerized ellipse method conferred better precision than trigonometric method for radiographs of femoral head (*p* < 0.01) (11).

Conventionally, using elliptical methods based on McLaren's equation mandates computerized equipment with corresponding software and the equipment is frequently in shortage in remote areas and developing countries. There is also a lack of picture archiving and communication system (PACS). In order to overcome these limitations, we invented a laser projector method utilizing ellipse (11), to measure the acetabular anteversion directly no matter the radiographs were in the PACS or were traditional plain films. Moreover, by using the laser projector, we can directly measure both the long and short axis of the acetabular cup as we adjusted the vertical holding distance between the projector and the plain film until the ellipse projected from the laser perfectly matched with the acetabular cup. The main purpose of our study is to examine the consistency and validity of the laser projector as compared to the Elliversion.

## Materials and methods

The aim of the present study is to investigate if the accuracy of measuring anteversion of acetabulum by the projector system is comparable to that by computerized ellipse method measuring software.

### Anteversion measurement

According to Dr Murray (13), *the operative anteversion* (OA) is the angle between the longitudinal axis of the patient and the acetabular axis as projected on to the sagittal plane; *the radiographic anteversion* (RA) is defined as the angle between the acetabular axis and the coronal plane; *the anatomical anteversion* (AA) is defined as the angle between the transverse axis and the acetabular axis when this is projected on to the transverse plane; and true and planar anteversion could be acquired by projecting the acetabular axis on to the transverse plane and to the plane that is perpendicular to coronal plane as well as acetabular plane, respectively. Based on our prior research (2), planar version is suitable for the evaluation of hip stability. Thus, in the present study, the mainstay measurement of acetabular anteversion is elliptical method, which concentrates on planar version.

**Figure 1:**
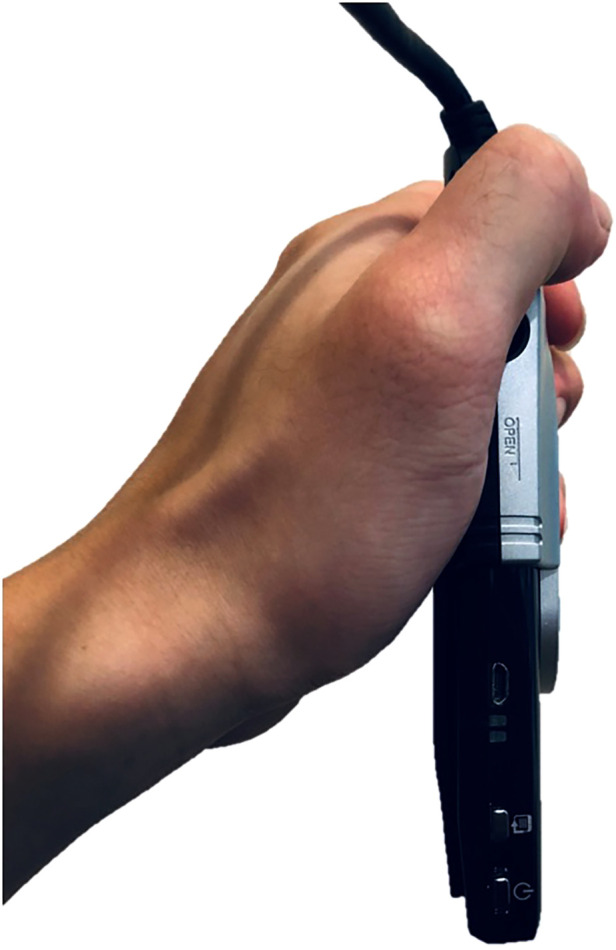
The portable laser projector that was used in the present study for the measurement of postoperative acetabular anteversion.

According to McLaren equation (14), planar version = arc sin (short axis/long axis), where short and long axes represented the axes of elliptical outline of the acetabular shell. However, a femoral head could easily eclipse the needed ellipse, resulting in difficult portraying. Therefore, we invented our own software, Elliversion, through which we could estimate the ellipse under most circumstances (11). Our newly designed laser projector method used the same elliptical method for measuring the anteversion as well, through converting computer-generated JPEG. frames with degree upon to projectable AVI. File, projected by any projector available in the market (Figure 1). Three modes have been designed, including Rudimentary mode: 1 degree per frame, Standard mode: 0.1 degree per frame and Precise mode: 0.05 degree per frame. The following data were analyzed through Precise mode.

### Measurement procedure

**Figure 2:**
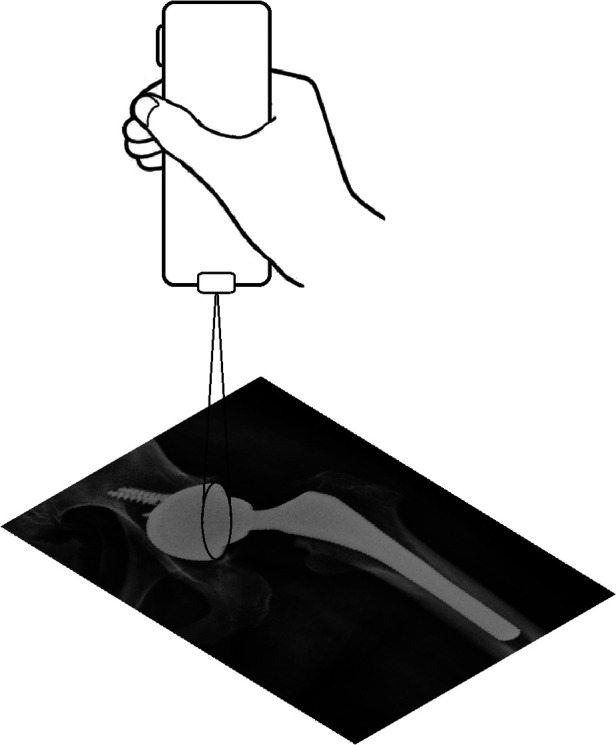
The portable laser projector held perpendicularly to the desired plain radiography as the distance between the projector and radiography adjusted until the ellipse projected from the laser perfectly matched with the cup.

**Figure 3:**
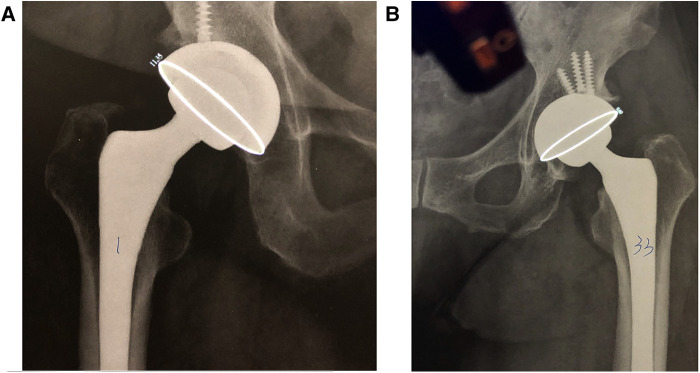
Post-operative radiographs of united total hip arthroplasty. Acetabular anteversion was measured using a portable laser projector. (**A**) A participant of right total hip arthroplasty with clear ellipse for measurement. With clear ellipse for the measurement, a degree of 11.35 anteversion was shown in this case following right total hip arthroplasty. (**B**) A participant of left total hip arthroplasty with a half of ellipse being obscured by femoral head, which is an example of poor-quality radiographs.

As demonstrated in the Figure 2, by using the laser projector, we can directly measure the long axis of the acetabular cup by holding the projector perpendicularly to the desired plain radiography as we adjusted the vertical distance between the projector and radiography until the long axis of the ellipse projected from the laser matched with the long axis of the cup. Once the AVI. file was played and the short axis of projected ellipse is perfectly matched with the cup, the measured anteversion will be shown on the film spontaneously [Figure 3A (11.35 degree in this case) and the video in the Supplement file 2].

### Study population

We collected 50 postoperative pelvis radiographs in anteroposterior (AP) view, which included acetabulum retrospectively. Eligible patients were aged 18 or older and had undergone either unilateral or bilateral THR. Those who suffered from hip fractures or congenital hip anomalies were excluded.

### Data collection

We measured every acetabular anteversion of the included 50 pelvic radiographs using both Elliversion and the laser projector. Initially, Author 1 completed measuring radiographs with Elliversion as the control group. Subsequently, two investigators (Author 1 and Author 2) independently used the projectors for measurements in two separate periods with 1-day intervals as the experimental group (Figure 3). Eventually, we measured 50 anteversion from Elliversion in control group and 200 from laser projectors in experimental group.

### Statistical synthesis

Our analyses comprised of intra- and inter-group comparison using two-sample student's *t*-test. Moreover, we calculated intraclass correlation coefficient (ICC) with two-way random effects model and the relationship of agreement to test the reliability. The ICC value of less than 0.5, between 0.5 and 0.75, between 0.75 and 0.9, and greater than 0.9 indicate poor, moderate, good, and excellent reliability (15). A *p*-value <0.05 was considered statistically significant. For intra-group comparison, we aimed to examine the “consistency” of our laser projector, to see if the measured results remain consistent when used by operators in different circumstances. On the other hand, regarding inter-group comparison, we scrutinized the “validity” of the projector, to see if there were statistical differences compared to Elliversion. In the presence of two operators, we determined that a target number of 49 patients would provide 90% power to detect a minimum ICC of 0.75 with expected ICC of 0.9 at a two-sided alpha level of 0.05 (16).

## Results

We included a total of 50 patients. Preoperative diagnosis included hip osteoarthritis, hip avascular necrosis, hip avascular necrosis with arthritis, and rheumatic arthritis (Table 1). All 50 postoperative pelvis AP radiographs with their corresponding anteversion measurement from both Elliversion and Laser projectors were presented in details in the Supplementary file. Among included radiographs, all acetabular cups are clear to identify.

**Table 1:** Patient demographic.

Age, mean (SD)	65.5 (10.9)
Male, *n* (%)	27 (54)
Operation site	
Right, *n* (%)	23 (46)
Left, *n* (%)	27 (54)
Preoperative diagnosis	
Hip osteoarthritis, *n* (%)	40 (80)
Hip avascular necrosis with arthritis, *n* (%)	3 (6)
Hip avascular necrosis, *n* (%)	6 (12)
Hip rheumatic arthritis, *n* (%)	1 (2)

SD, standard deviation.

The intra-group reliability was summarized in Table 2. Laser A1 and A2 represented the mean anteversions measured by Author 1 in the first and second time, respectively, with a certain interval of period. There was no significant difference in measurements between A1 and A2 (MD, 0.25; 95% CI, −0.10 to 0.61; *p* = 0.15), with the ICC value of 0.976 (95% CI, 0.958 to 0.986), suggesting excellent intra-observer reliability. On the other hand, Laser B1 and B2 accounted for mean measurements acquired by Author 2 in two independent periods. The difference between B1 and B2 was little (MD, 0.18; 95% CI, −0.05 to 0.42; *p* = 0.12), with the ICC value of 0.988 (95% CI, 0.98 to 0.993), indicating excellent intra-observer reliability as well. Moreover, Laser A and B were the mean anteversion of A1, A2 and B1, B2. The results acquired by two investigators (A vs. B) were comparable (MD, −0.12; 95% CI, −0.52 to 0.27; *p* = 0.54), with excellent inter-rater reliability (ICC, 0.967; 95% CI, 0.949 to 0.979).

**Table 2:** Laser B1 represents the first measurement by the Author 2; Laser B2 represents the "second" measurement by the Author 2.

Mean	SD	Mean	SD	MD	95% CI	*p*-value
Laser A1		Laser A2		A1 vs. A2		
15.49	5.92	15.24	5.95	0.25	[−0.10 to 0.61]	0.15
ICC (95% CI): 0.976 (0.958 to 0.986)				*p* = 2.07e-34		
Laser B1		Laser B2		B1 vs. B2		
15.58	5.79	15.39	5.97	0.18	[−0.05 to 0.42]	0.12
ICC (95% CI): 0.988 (0.98 to 0.993)				*p* = 1.51e-42		
Laser A		Laser B		A vs. B		
15.36	5.91	15.49	5.82	−0.12	[−0.52 to 0.27]	0.54
IRR (95% CI): 0.967 (0.949 to 0.979)				*p* = 1.49e-97		

SD, standard deviation; CI, confidence interval; Laser A1 represents the first measurement by the Author 1; Laser A2 represents the second measurement by the Author 1; Laser B1 represents the first measurement by the Author 2; Laser B2 represents the first measurement by the Author 2; ICC, intraclass correlation coefficient; IRR, inter-rater reliability.

The comparison between Elliversion and Laser projector was outlined in Table 3. The estimated effect in the anteversion measurement between two Elliversion and Laser were similar (MD, −0.17; 95% CI, −0.38 to 0.04; *p* = 0.12), with the ICC level of 0.984 (95% CI, 0.971 to 0.991), indicating excellent reliability.

**Table 3:** Comparison between Elliversion and Laser projector method.

Elliversion		Laser		Elliversion vs. Laser		
Mean	SD	Mean	SD	MD	95% CI	*p*-value
15.26	5.84	15.42	5.82	−0.17	[−0.38 to 0.04]	0.12
ICC (95% CI): 0.984 (0.971 to 0.991)				*p* = 2.57e-34		

SD, standard deviation; CI, confidence interval; ICC, interclass correlation coefficient.

## Discussion

Due to advanced surgical techniques and increased endurance of hip prostheses, the long-term survival after THR has improved a lot. The primary reason for hip reoperation falls into instability, dislocation and mechanical loosening (17, 18). Although the causes of dislocation are multifactorial, the most recognized one is associated with acetabular orientation, including inclination and anteversion. Erroneous orientation will result in femoroacetabular impingement syndrome (FAI) leading to dislocation (19). Inadequate and excessive anteversion of acetabulum will increase the respective risk of anterior impingement in sitting position and posterior impingement in standing position (19). On the other hand, a high inclination of hip cup could possibly lead to FAI by posing detrimental edge-loading and contact pressure that accelerated overall wear (20). Though the best orientation of acetabulum is still debated with Charnley et al. (21) suggesting no absolutely perfect value, an angle between 15–30 degree (22, 23) for anteversion and 40–55 degree (24, 25) for inclination are generally recommended. Compared to acetabular anteversion, the measurement of inclination could be easily achieved on plain radiographs. As a consequence, precisely measuring postoperative anteversion is challenging and crucial in patients receiving THR.

Over the past decades, computer-aided diagnosis has evolved rapidly and grown from a state of infancy to certain maturity (26). To date, there are various computer-aided 2D methods for directly measuring postoperative acetabular anteversion upon plain radiographs and each method carried its own advantages and disadvantages. Although there is still no gold-standard measurement of acetabular orientation, a variety of methods have been proposed to identify the postoperative position, such as methods of Lewinnek et al. (4), Widmer et al. (10), Hassan et al. (27), Ackland et al. (3), Woo et al. (28) and Liaw et al. (2). Among these verified methods, Nho et al. (29) compared their accuracy utilizing CT as a reference standard and found out that Lewinnek et al., Hassan et al., Liaw et al., and Woo et al., possessed higher reliability and validity. Furthermore, Park and colleagues (30) concluded that the method proposed by Liaw et al. was more accurate than others while utilizing plain films for measuring the acetabular anteversion after THR, with the PolyWare programme as the reference standard. In fact, Liaw's team has created many new modalities in pursuit of better precision and convenience. The primary concept of Liaw et al. that was compared in Park's study was trigonometric, which would be an ideal method for measuring radiographs without femoral heads. However, for those with femoral heads, it is more appropriate to utilize elliptical method which conferred better precision if femoral heads were included because the outline of the shell tends to be obscured (11). Moreover, during our daily practice, it is almost inevitably to include femoral heads in the postoperative radiographs. As a result, we selected the ellipse as the measuring basis in our laser projector method.

The present study successfully identified the consistency and validity of our newly designed laser projectors, demonstrating real-time, intuitive, and convenient product design comparing to Elliversion. Although we identified good correlation between Elliversion and the laser projector, the projector is operator-dependent, which carried a certain risk of the dissociation of these two methods. Of note, the most commonly encountered factor is operating instability caused by inadequate hand-eye coordination due to improficiency, resulting in the imprecision of measurement. Therefore, it is inevitable for novices to have individual learning curves before they can attain the highest precision.

We found that there was no significant difference in measured anteversion between Elliversion and the projector. In addition, distinctions in two separate operators utilizing the projector were negligible. Some orthopedic surgeons may question the necessity of this projector in the presence of Elliversion. Indeed, in most developed countries or modern cities where hospitals are equipped with PACS, the projector is superfluous. However, in hospitals located in developing countries or in the remote regions that are either geographically isolated, socioeconomically unequal or of indigenous health inequity, PACS could be luxurious. In addition, one of the eight key features of remote medical practice, “increased clinical acumen”, mandates remote doctors to have high level of clinical acumen to make the diagnosis and cope with the diseases because hospitals where they are practicing often lack of diagnostic support (31), not to mention PACS. Thus, we believe that our projector method are able to aid clinical acumen whenever doctors confront patients with operation history of THR in remote areas.

## Conclusion

We invented a portable laser projector method utilizing the elliptical method for orthopedic surgeons to feasibly and conveniently measure postoperative acetabular anteversion. Our study reported the consistency and validity of this laser projector as there is no significant difference between Elliversion and Laser projector, notably with excellent intra- and inter-observer reliability, demonstrating real-time, intuitive, and convenient product design comparing to Elliversion. Most importantly, we look forward to helping elevate clinical acumen when doctors provide care to patients after THR, especially in remote areas.

## Data Availability

The original contributions presented in the study are included in the article/**Supplementary Material**, further inquiries can be directed to the corresponding author/s.
