# Supplementary material for: Laser projector method for measuring postoperative acetabular anteversion after total hip replacement
Source: Front Surg. 2022 Oct 24;9:1033453. doi: 10.3389/fsurg.2022.1033453 (PMC9637855; doi:10.3389/fsurg.2022.1033453)
Supplement: Supplementary file 2 [file Datasheet1.pdf]

Supplementary File

Supplementary File 1:

50 postoperative pelvis radiographs in AP view

Supplementary File 2:

Video link used in the present study for the measurement of postoperative acetabular anteversion

Video Legend:

Video of Precise mode (0.05 degree per frame) used in the present study for the measurement of postoperative acetabular anteversion.

Supplementary File 1

50 postoperative pelvis radiographs in AP view

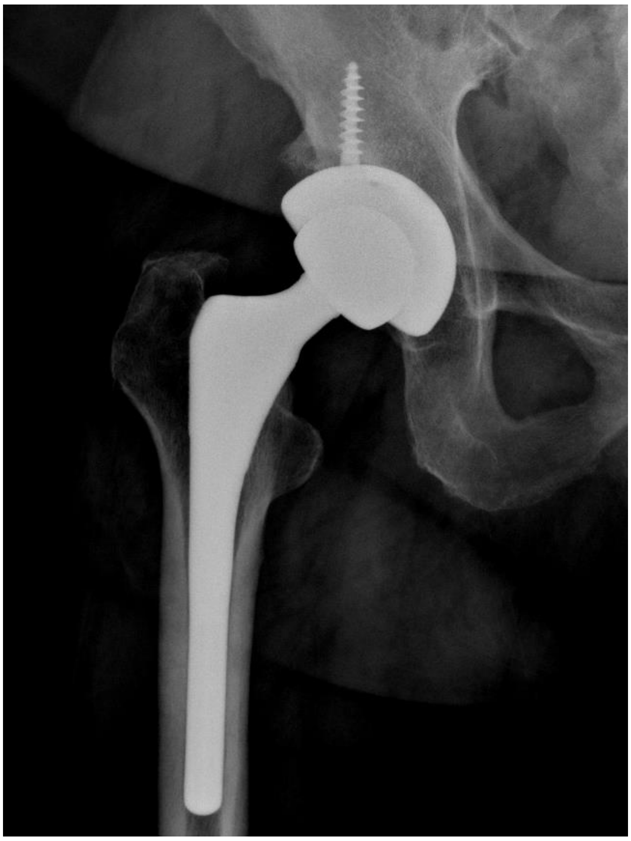

Case 1

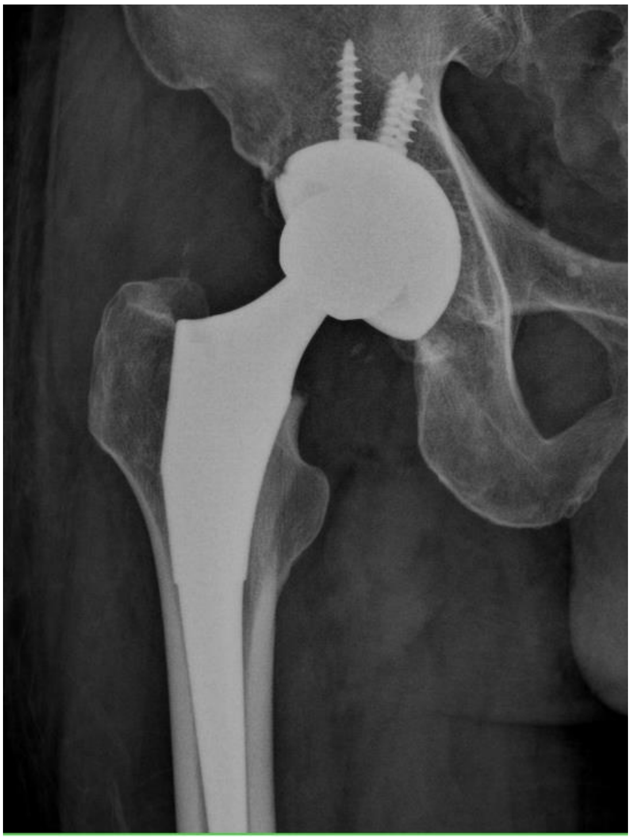

Case 2

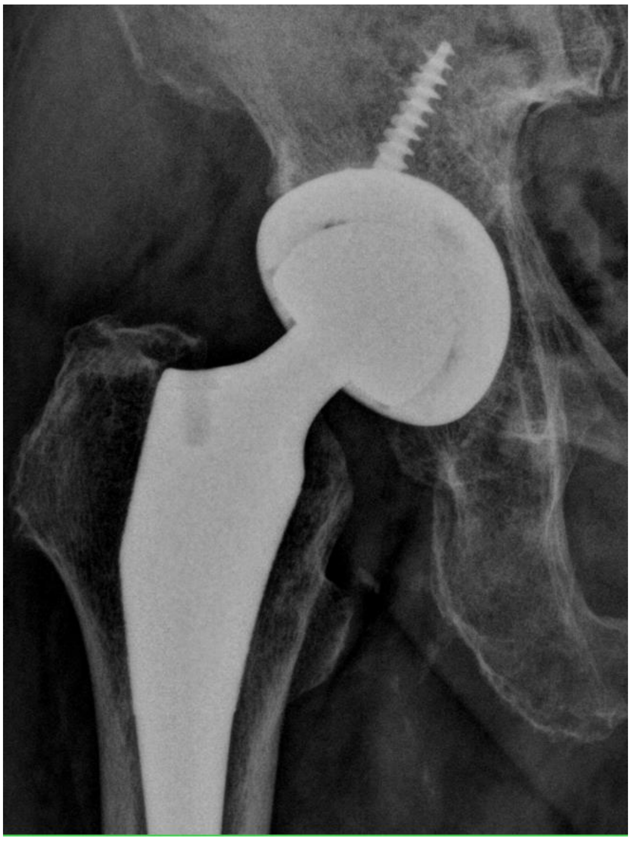

Case 3

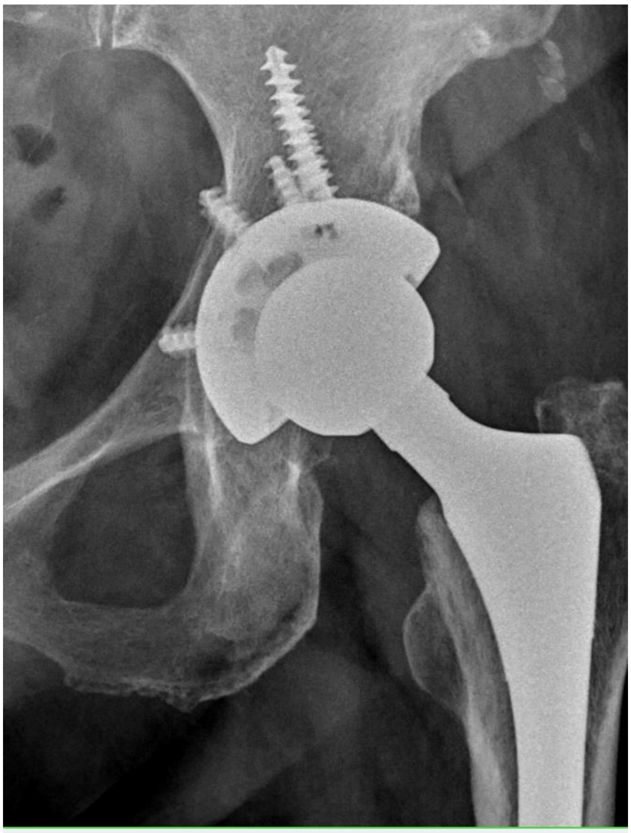

Case 4

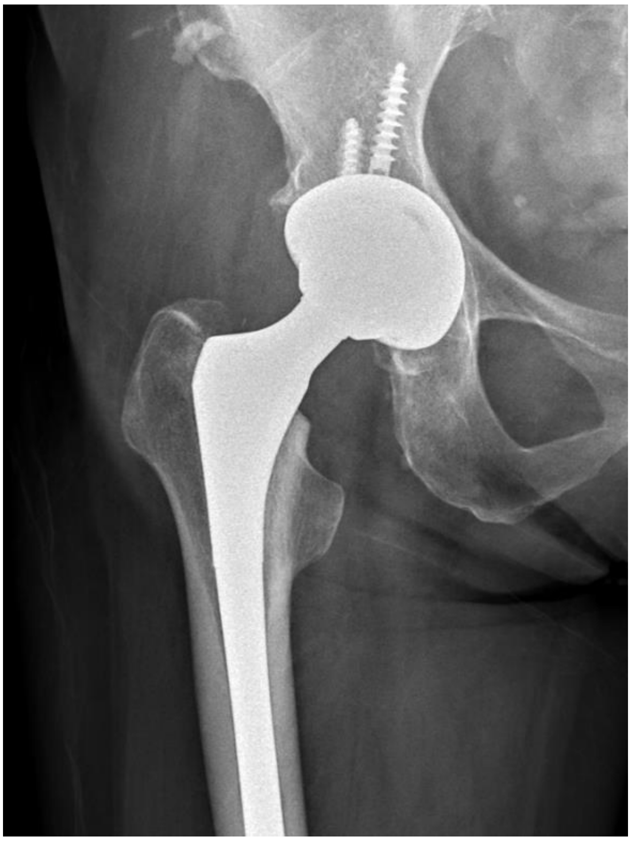

Case 5

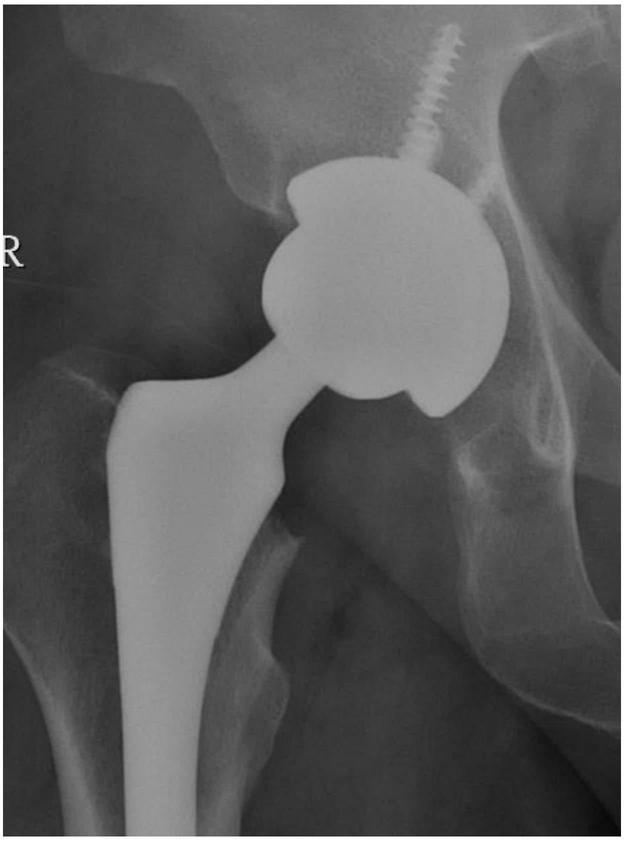

Case 6

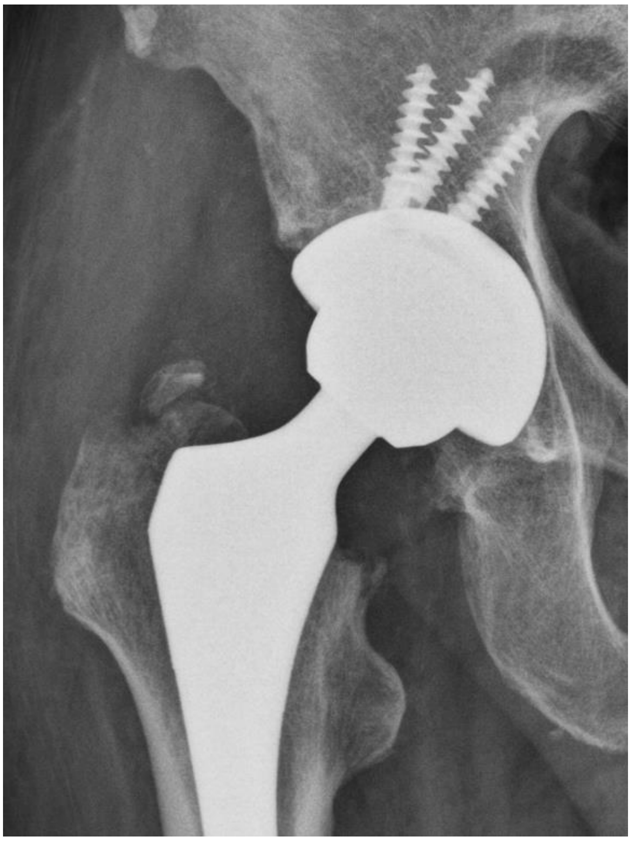

Case 7

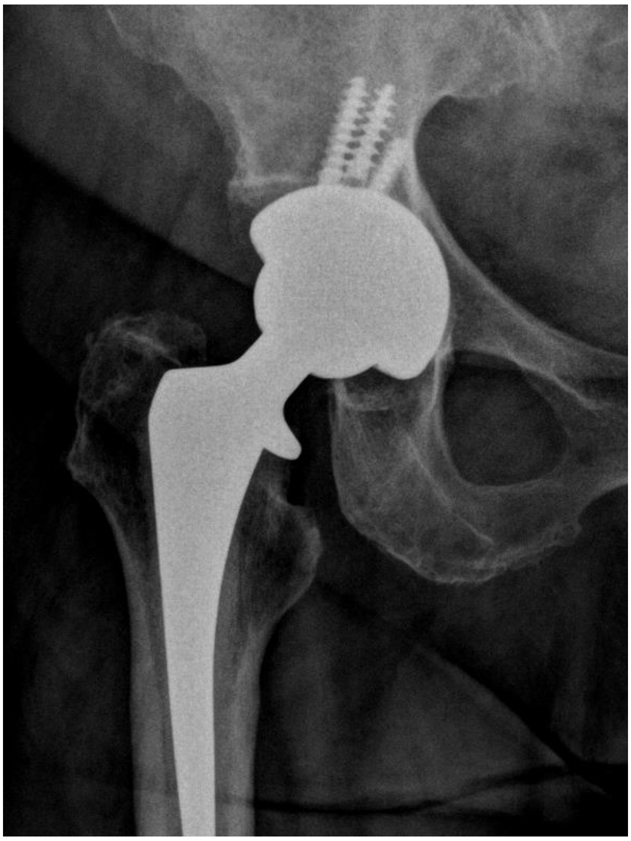

Case 8

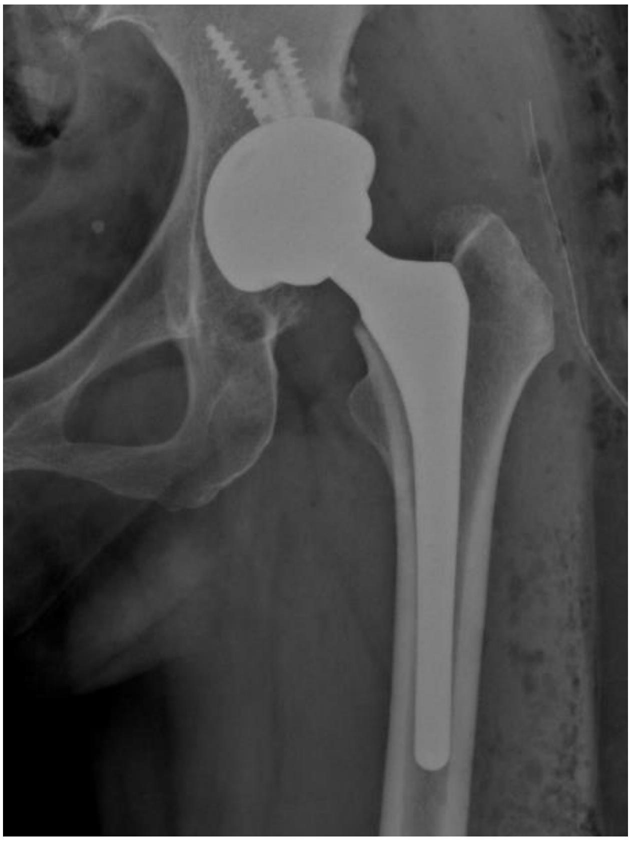

Case 9

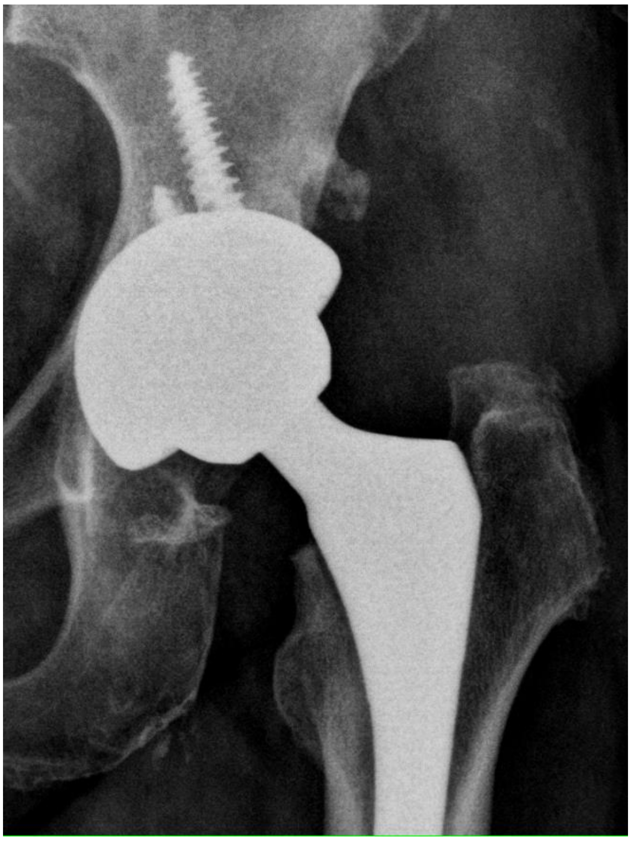

Case 10

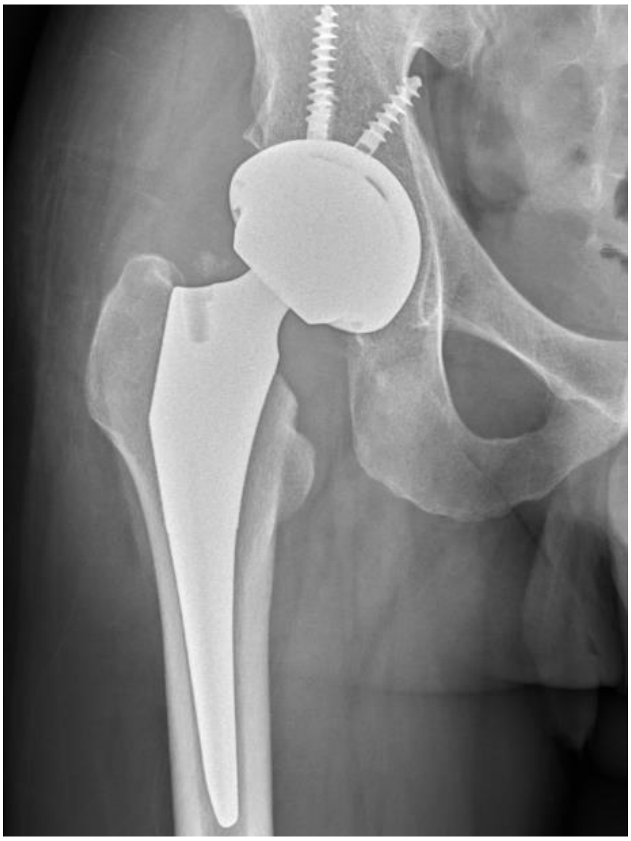

Case 11

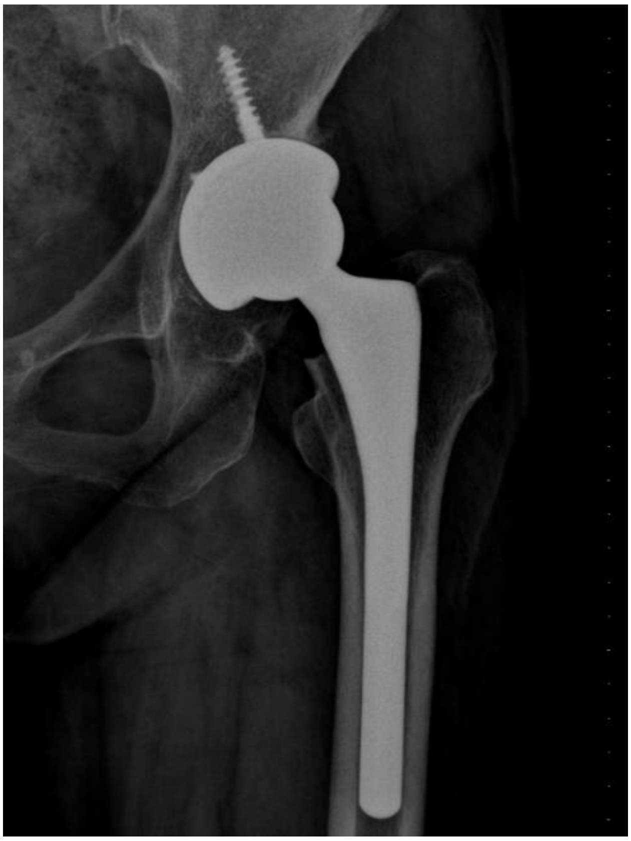

Case 12

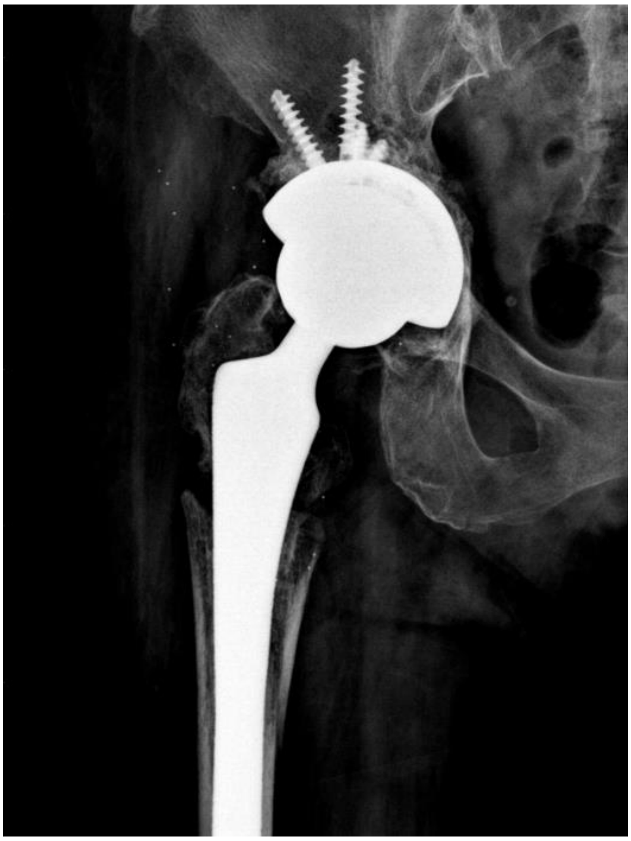
Case 13

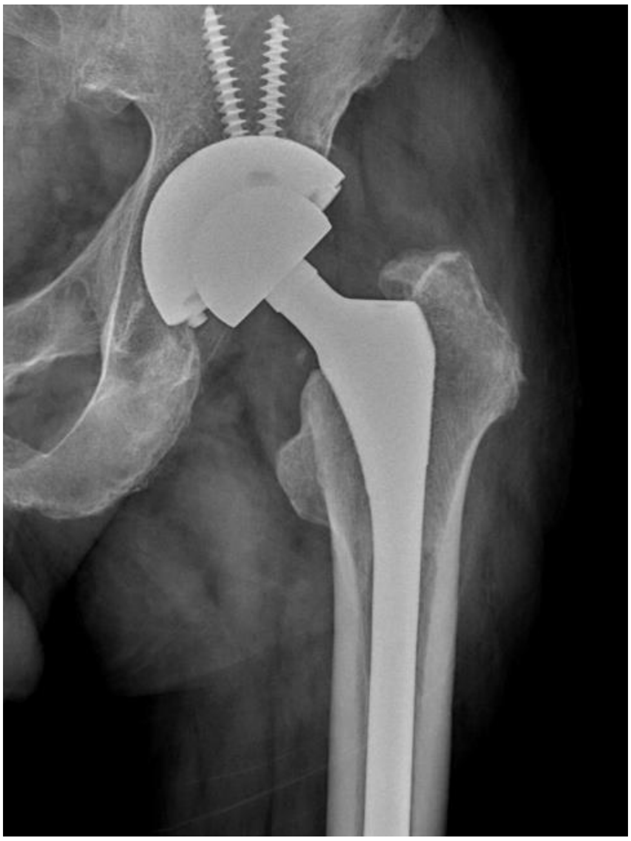
Case 14

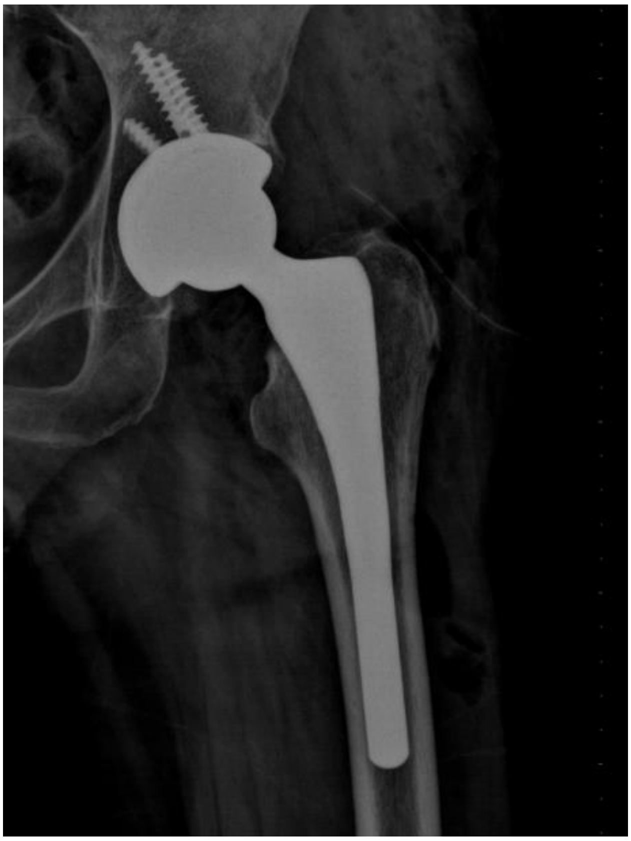

Case 15

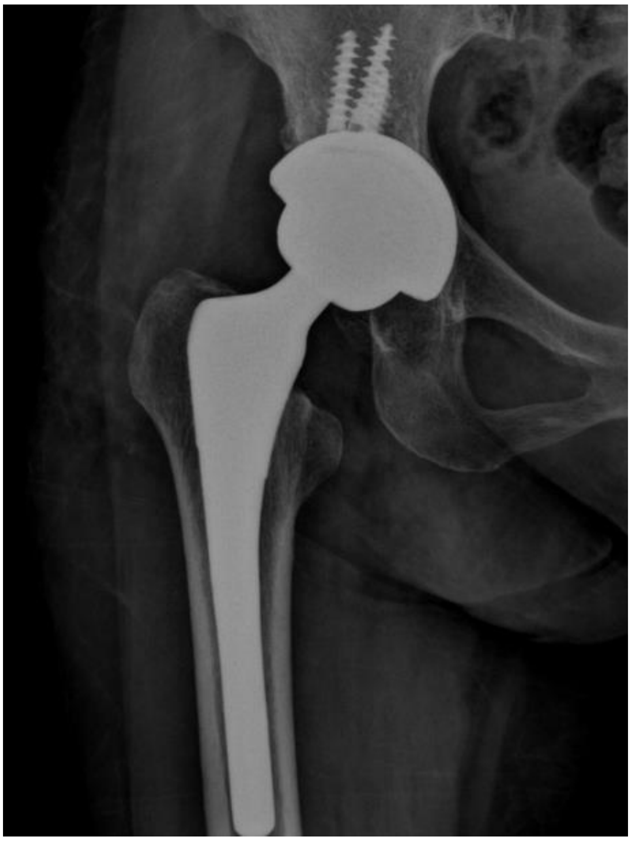

Case 16

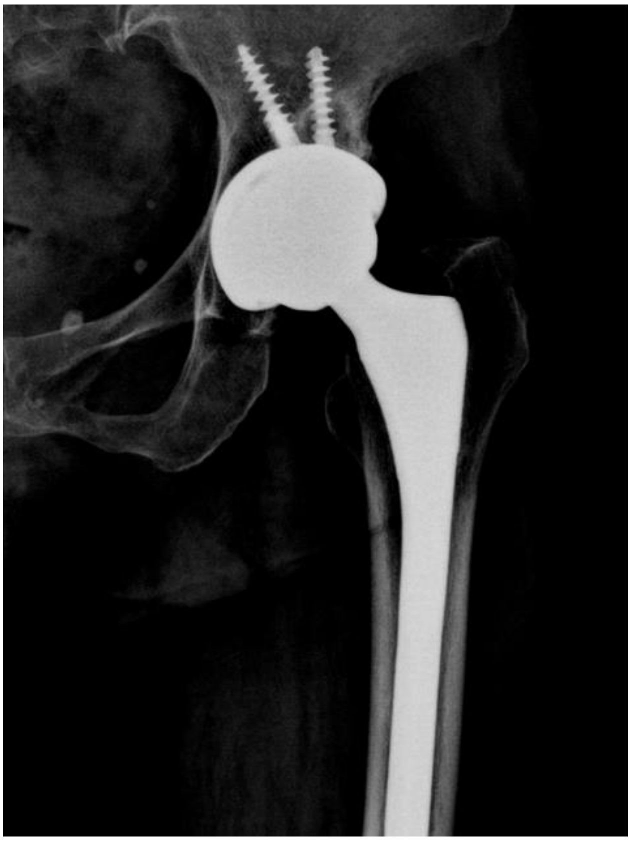

Case 17

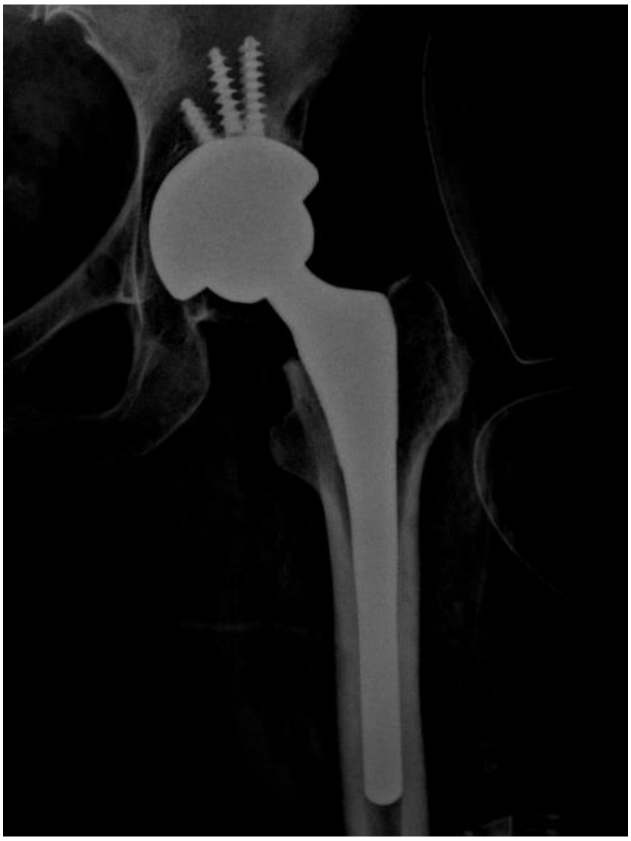

Case 18

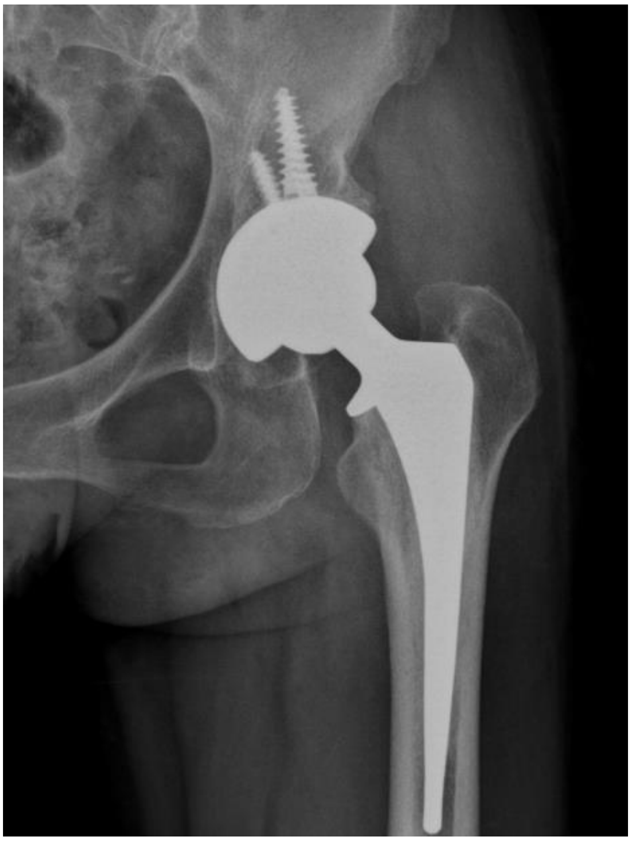

Case 19

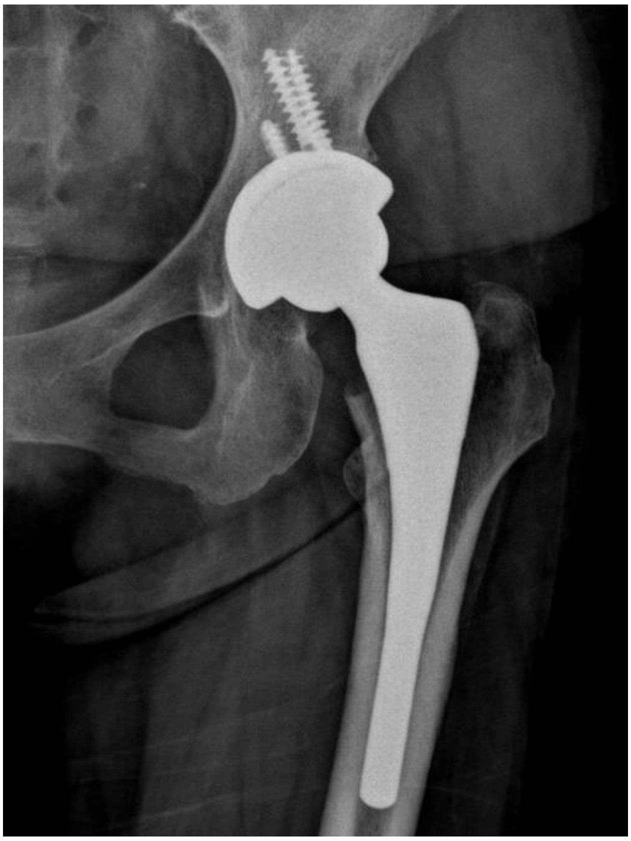

Case 20

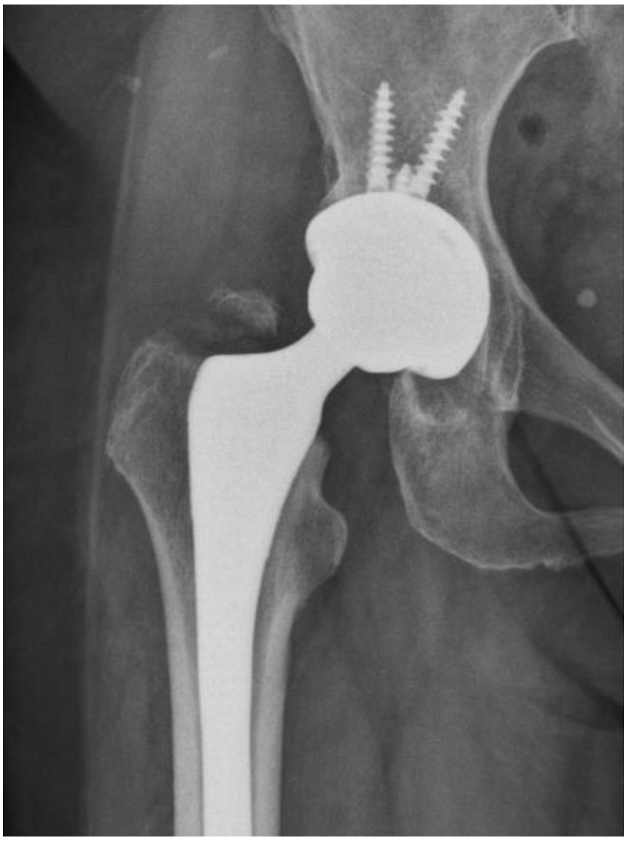

Case 21

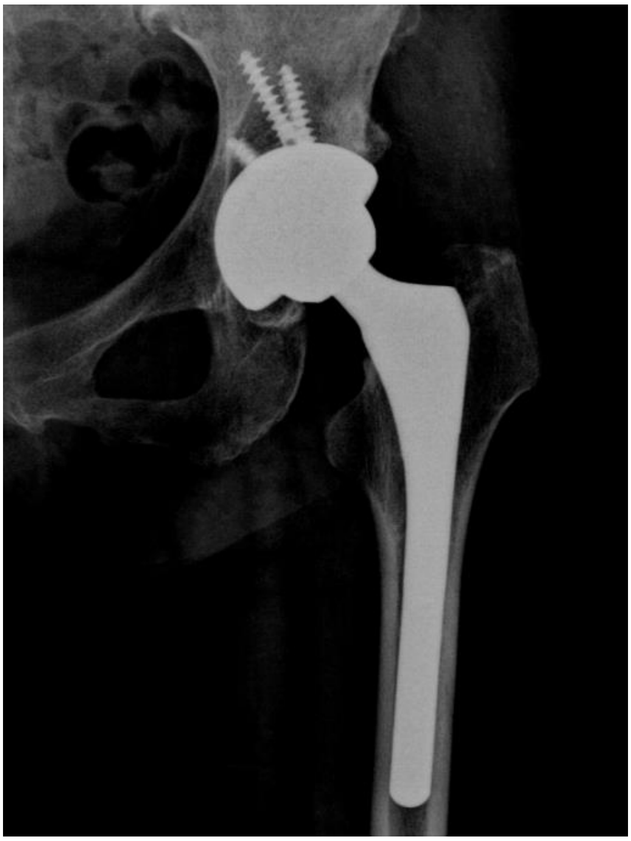

Case 22

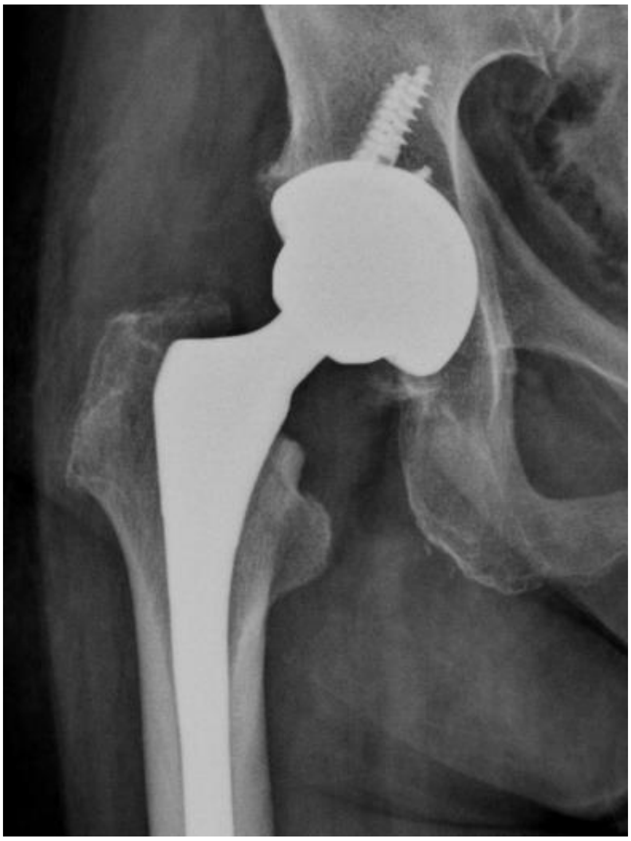

Case 23

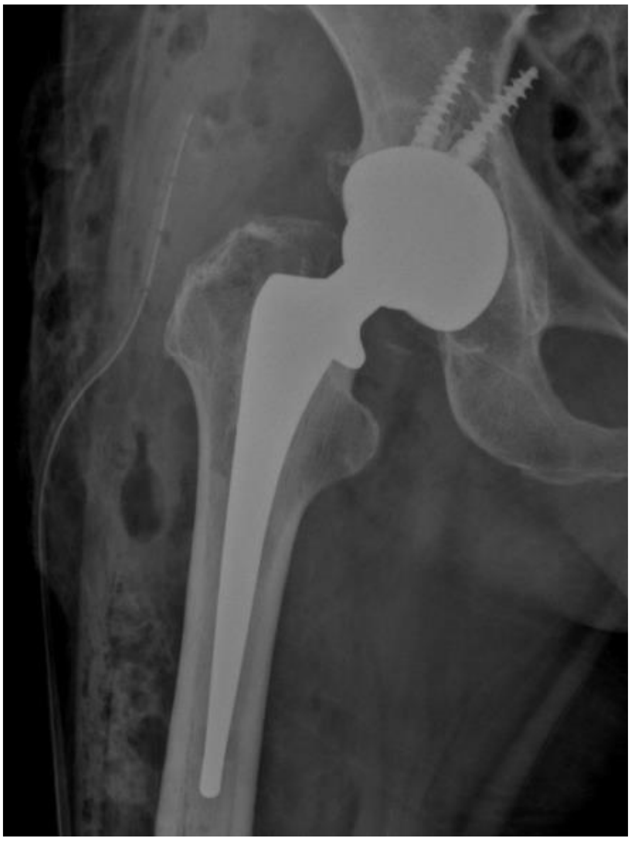

Case 24

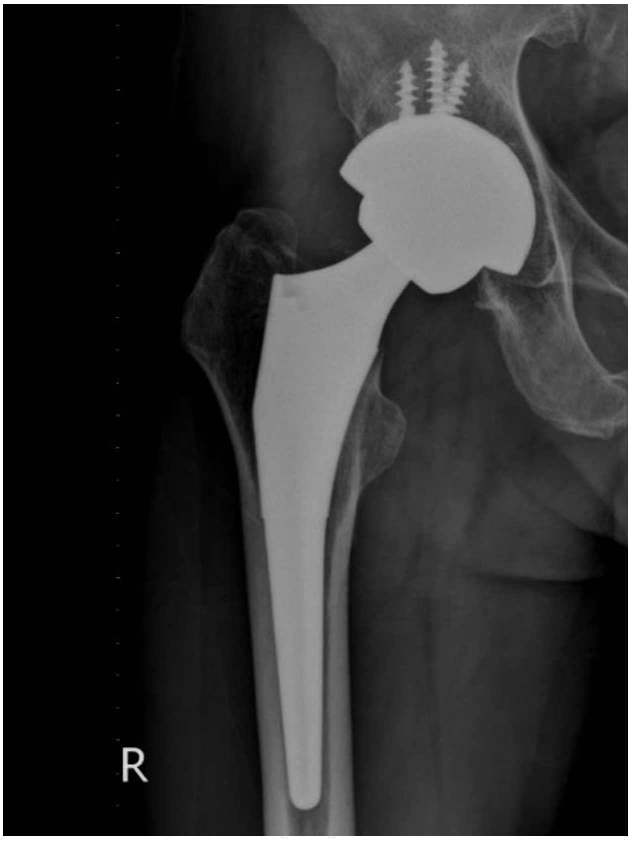

Case 25

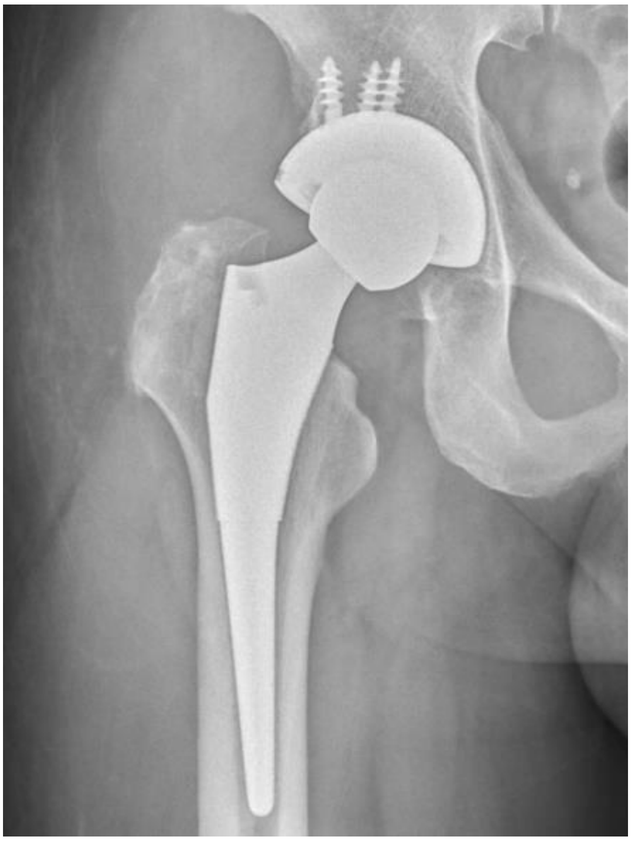

Case 26

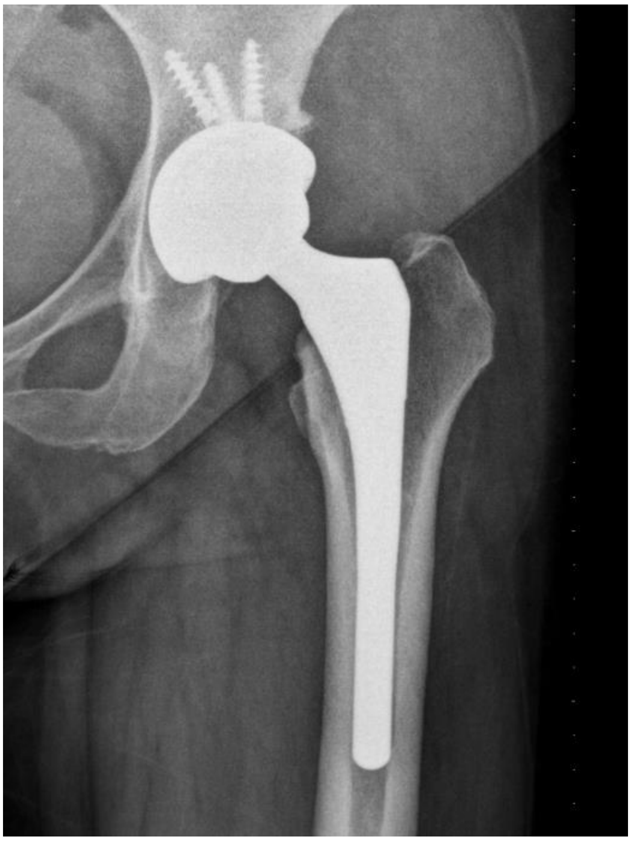

Case 27

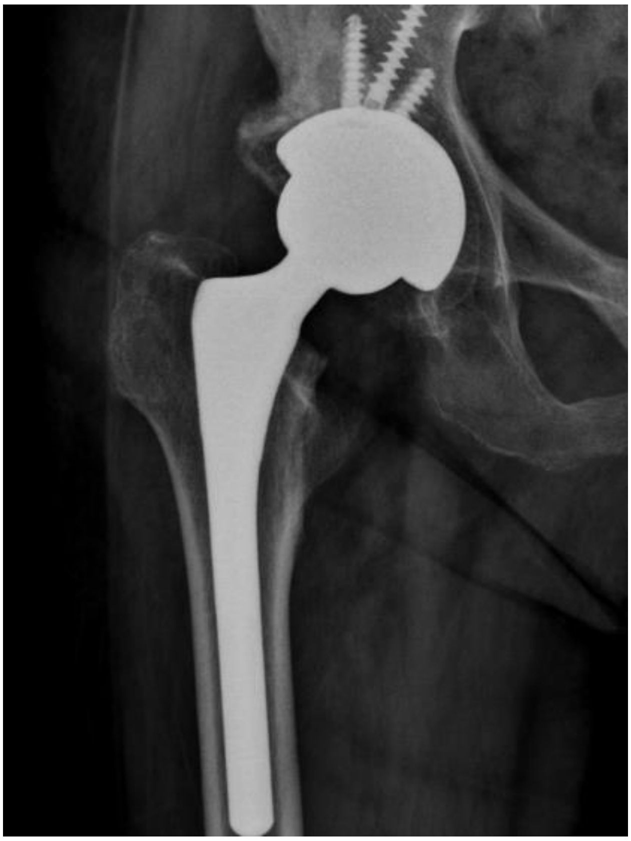

Case 28

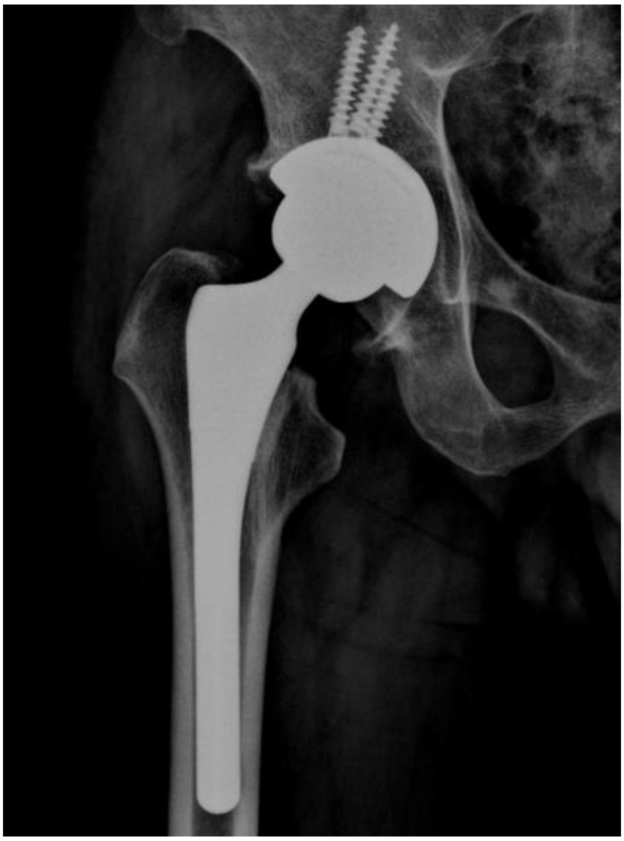

Case 29

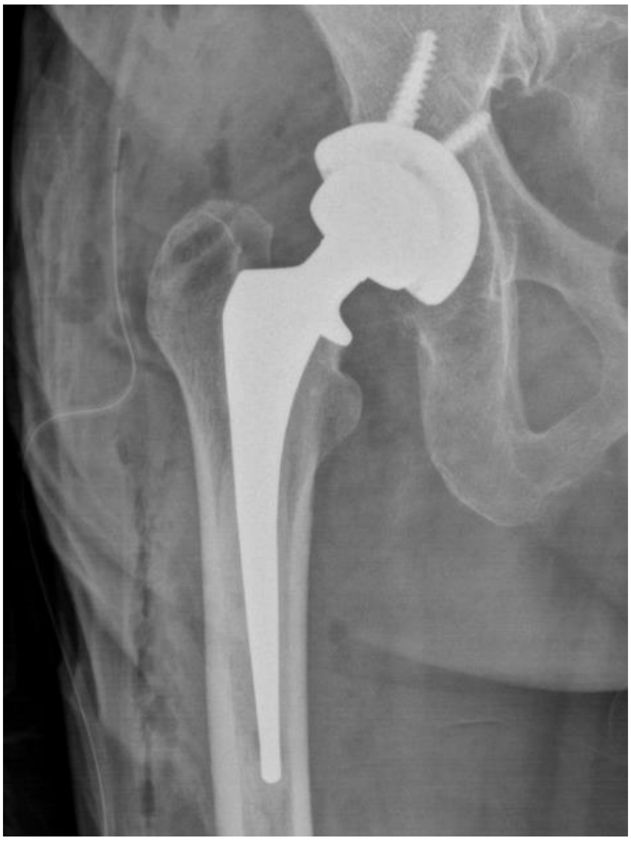

Case 30

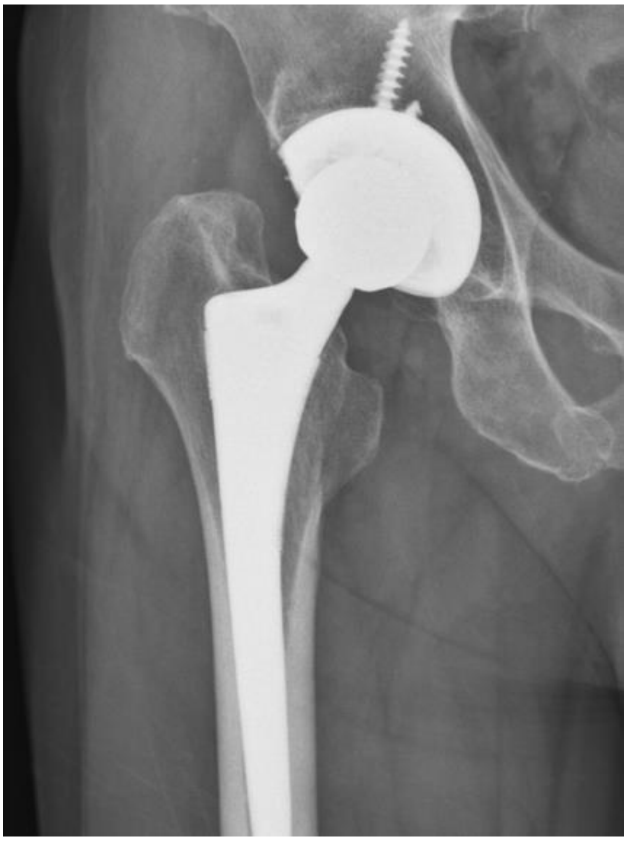
Case 31

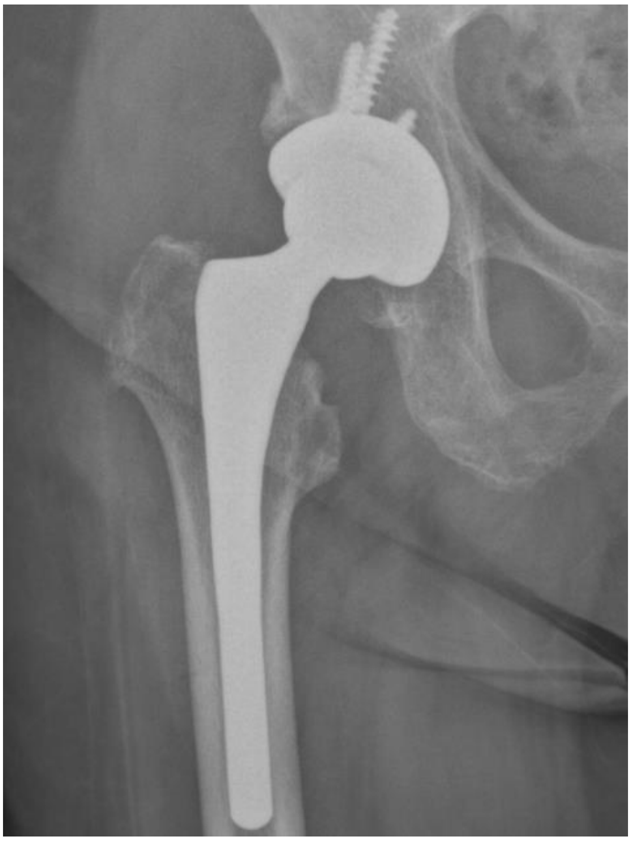
Case 32

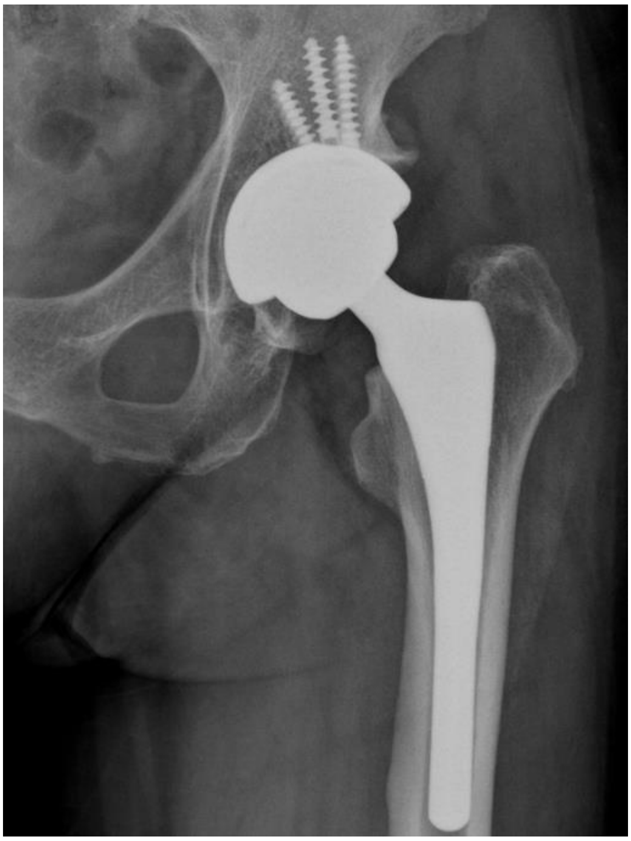

Case 33

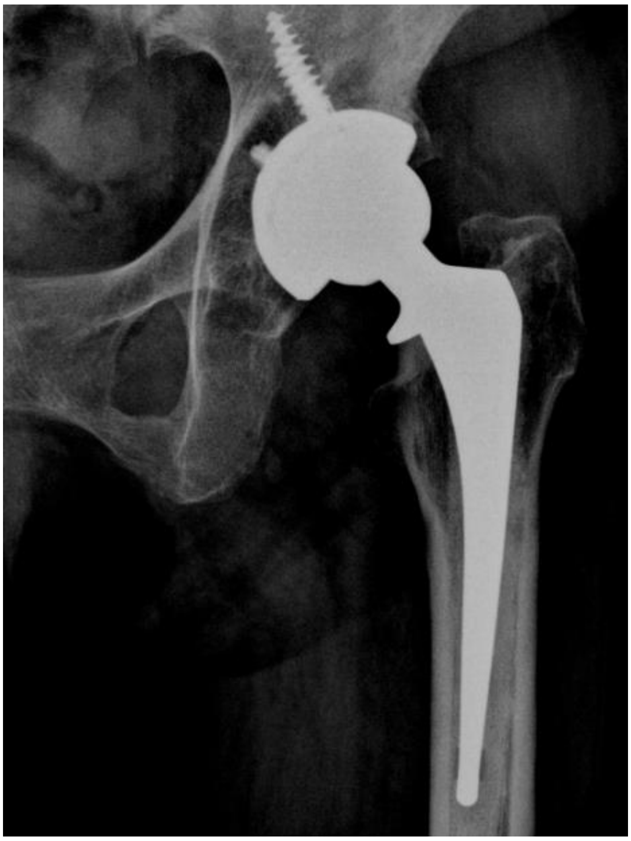

Case 34

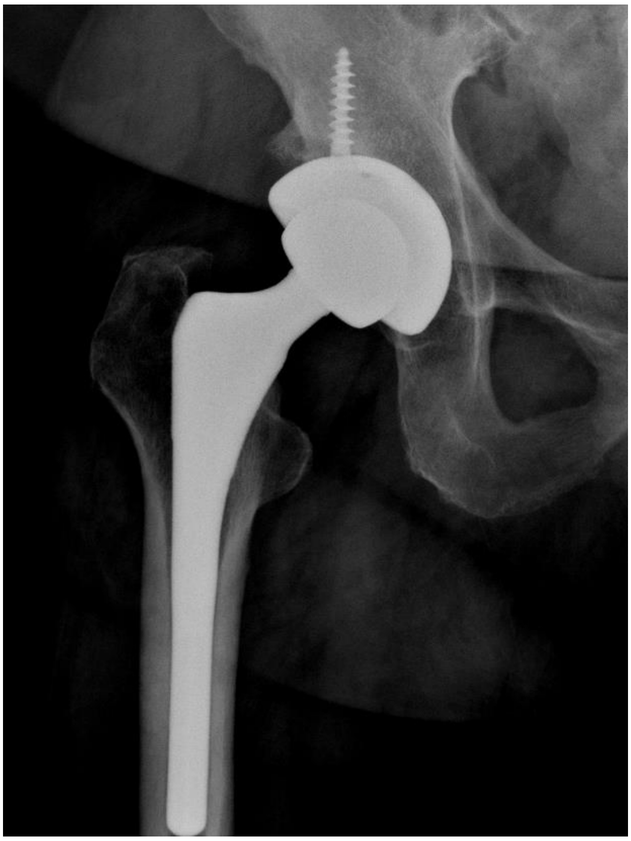

Case 35

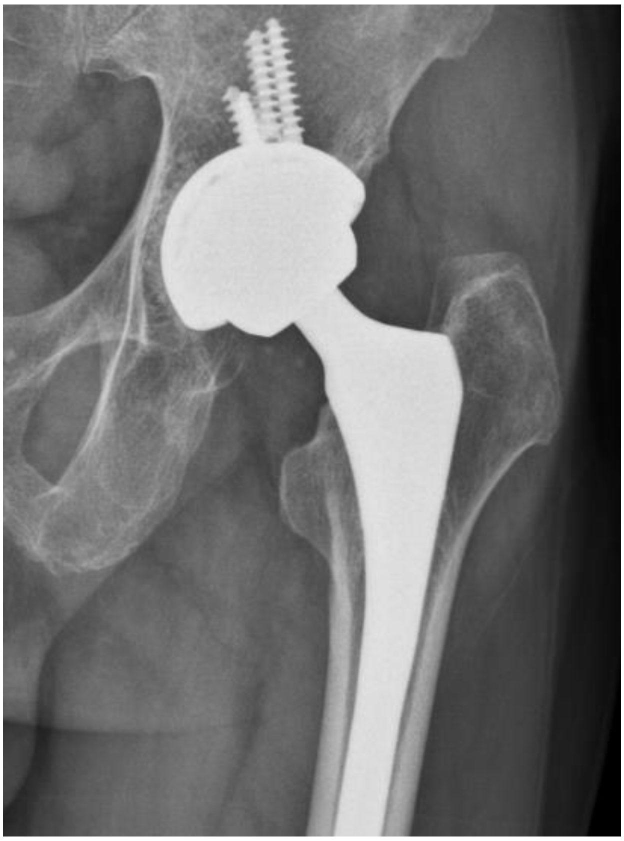

Case 36

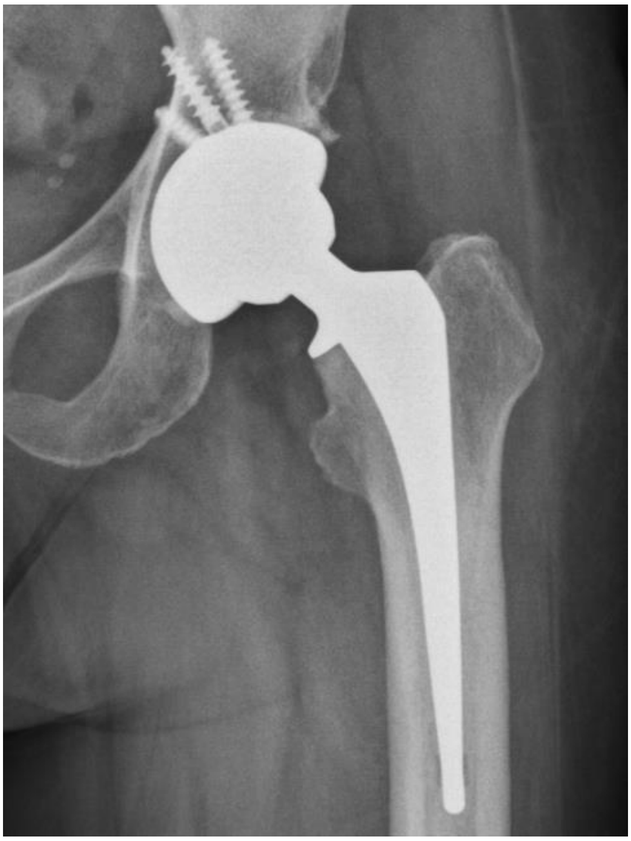

Case 37

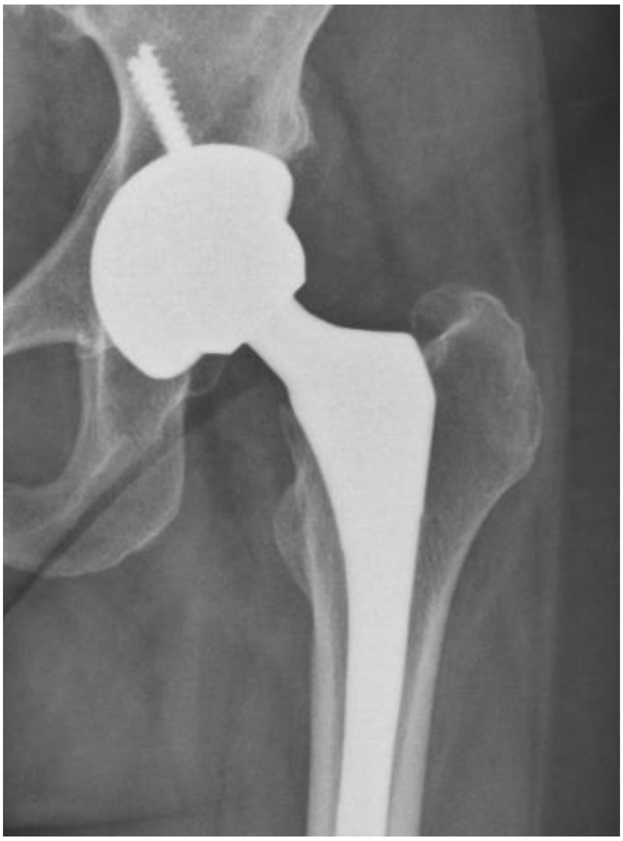

Case 38

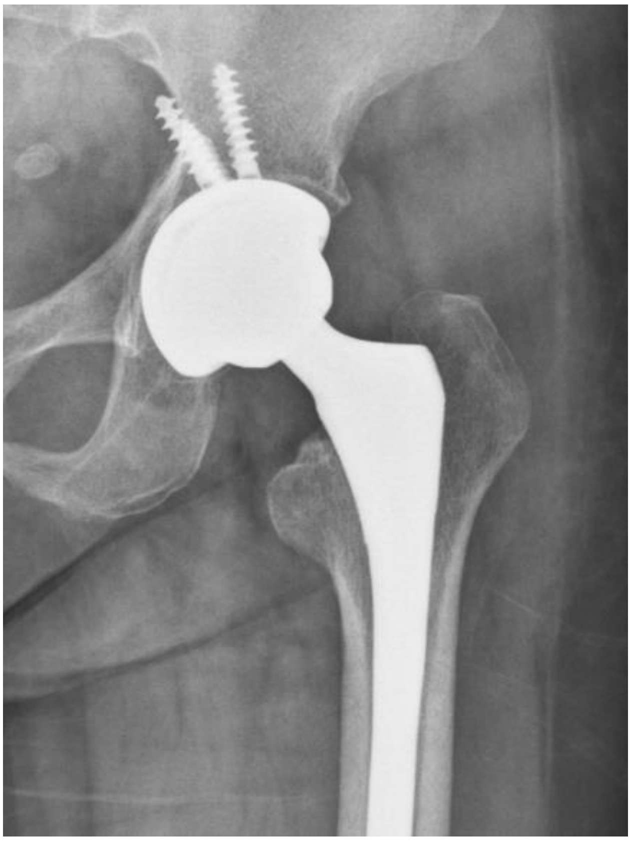

Case 39

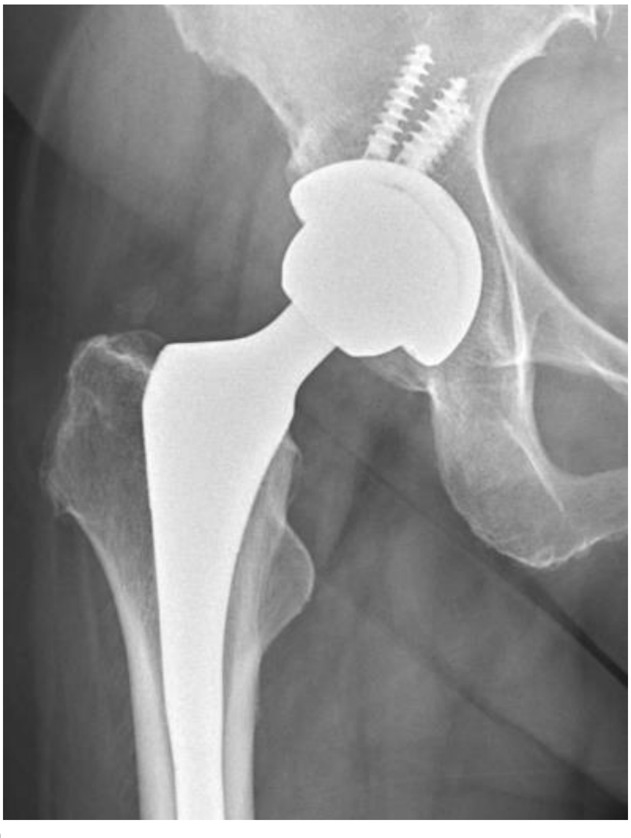

Case 40

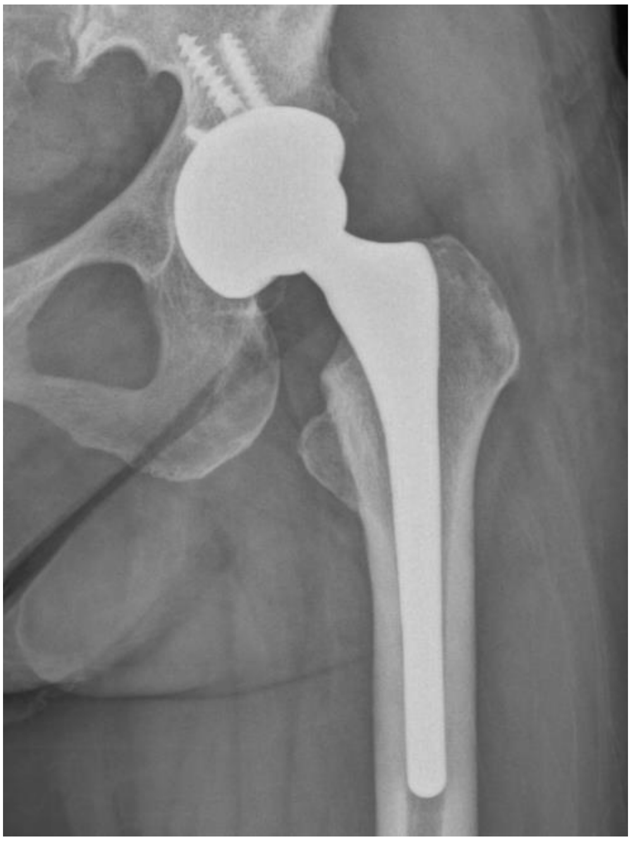

Case 41

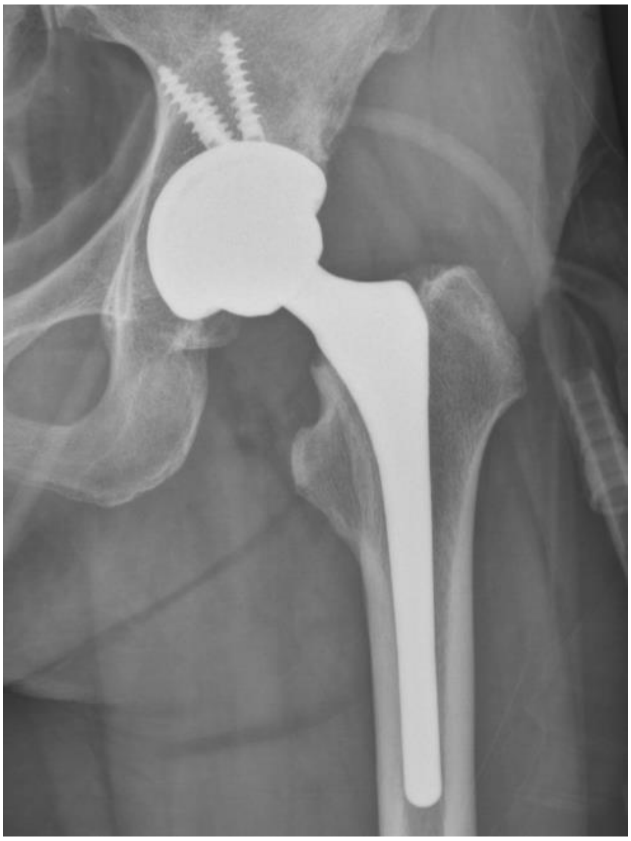

Case 42

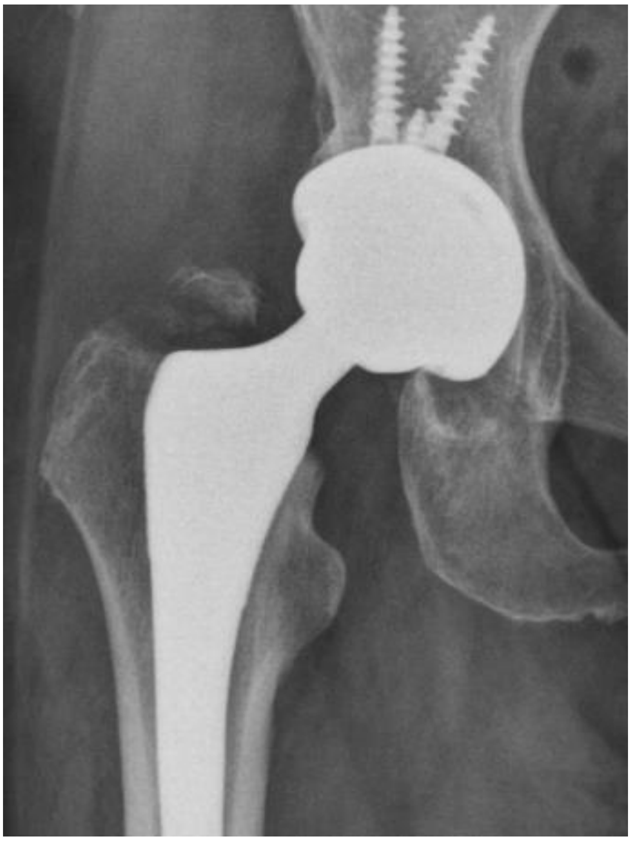
Case 43

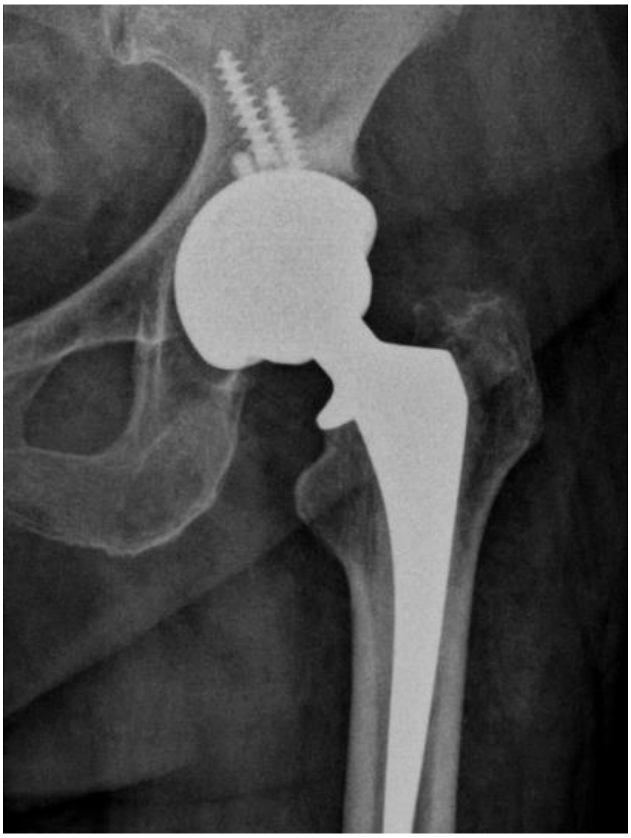
Case 44

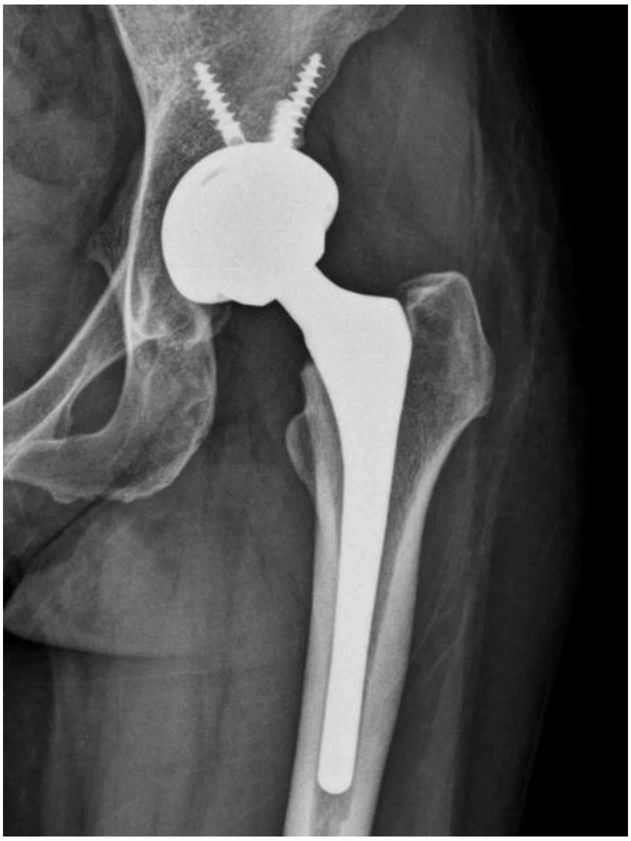

Case 45

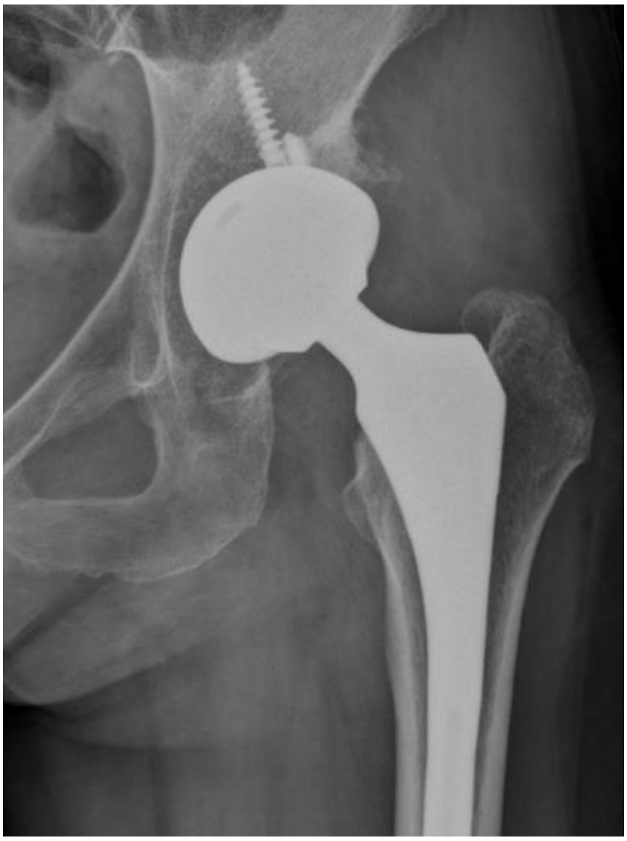

Case 46

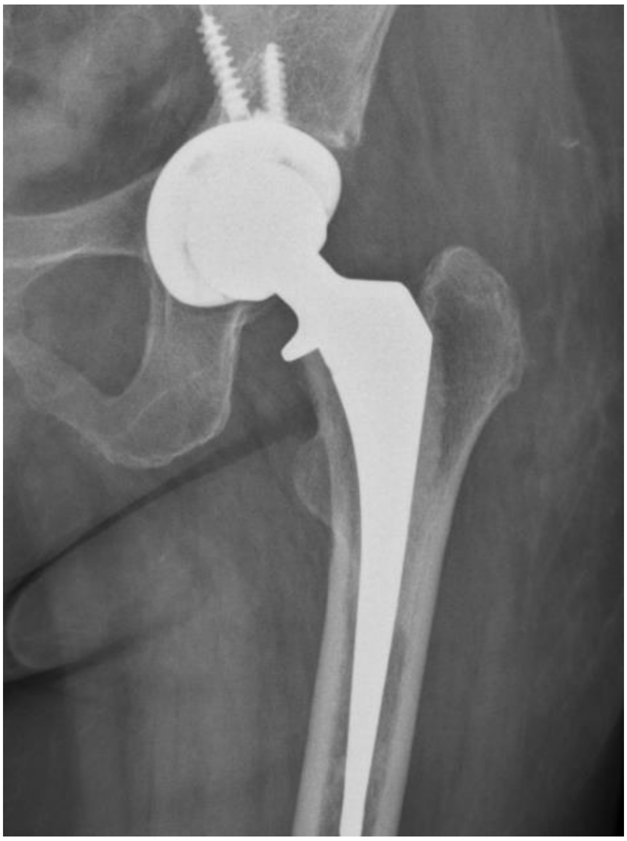

Case 47

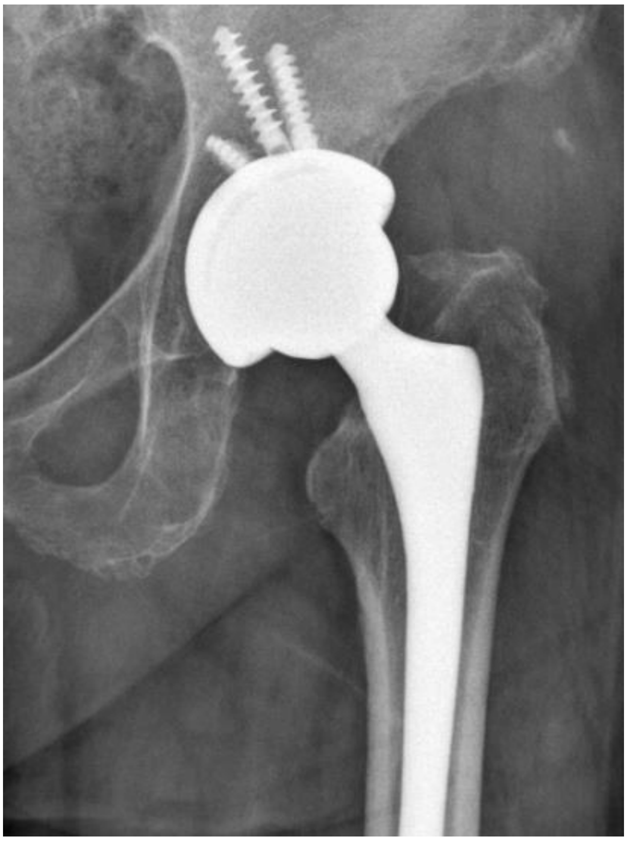

Case 48

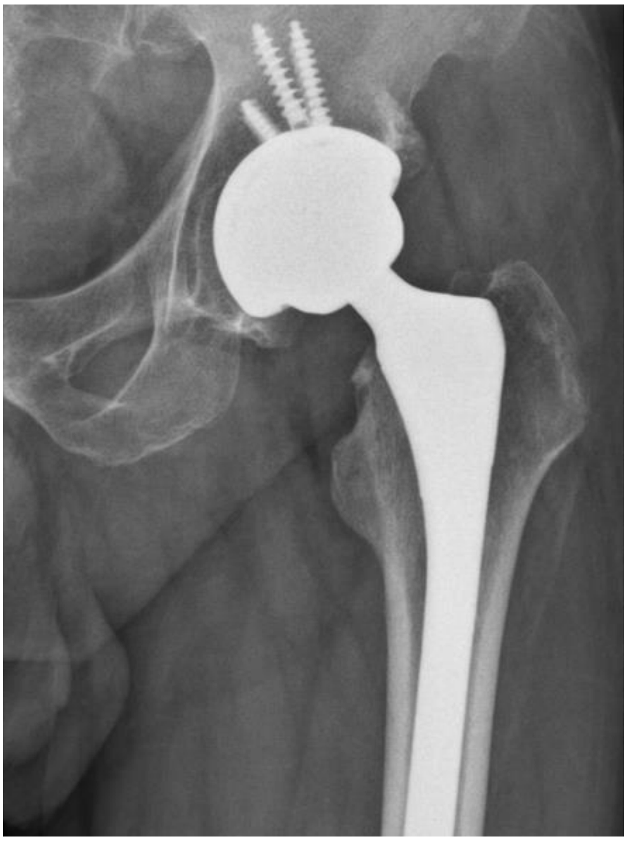

Case 49

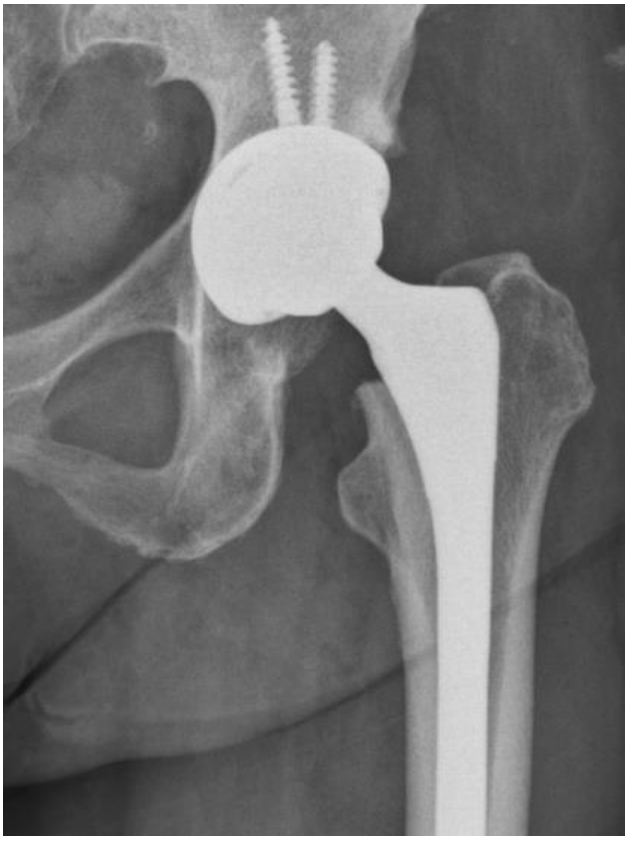

Case 50

Supplementary File 2

Video link used in the present study for the measurement of postoperative acetabular anteversion

https://www.youtube.com/watch?v=Y9R3ZAzgm4I&feature=youtu.be

Video Legend:

Video of Precise mode (0.05 degree per frame) used in the present study for the measurement of postoperative acetabular anteversion.
